# Early prediction of Alzheimer’s disease using artificial intelligence and cortical features on T1WI sequences

**DOI:** 10.3389/fneur.2025.1552940

**Published:** 2025-03-12

**Authors:** Rong Zeng, Beisheng Yang, Faqi Wu, Huan Liu, Xiaojia Wu, Lin Tang, Rao Song, Qingqing Zheng, Xia Wang, Dajing Guo

**Affiliations:** ^1^Department of Radiology, the Second Affiliated Hospital of Chongqing Medical University, Chongqing, China; ^2^Department of Radiology, the Third Affiliated Hospital of Chongqing Medical University, Chongqing, China; ^3^Department of Medical Service, Yanzhuang Central Hospital of Gangcheng District, Jinan, China; ^4^GE Healthcare, Shanghai, China; ^5^Department of Radiology, Chongqing University Cancer Hospital, Chongqing, China; ^6^Department of Radiology, Chongqing Western Hospital, Chongqing, China

**Keywords:** Alzheimer’s disease, mild cognitive impairment, prediction, gray matter, network, radiomics, magnetic resonance imaging

## Abstract

**Background:**

Accurately predicting the progression of mild cognitive impairment (MCI) to Alzheimer’s disease (AD) is a challenging task, which is crucial for helping develop personalized treatment plans to improve prognosis.

**Purpose:**

To develop new technology for the early prediction of AD using artificial intelligence and cortical features on MRI.

**Methods:**

A total of 162 MCI patients were included from the Alzheimer’s Disease Neuroimaging Initiative (ADNI) database. By using a 3D-MPRAGE sequence, T1W images for each patient were acquired. All patients were randomly divided into a training set (*n* = 112) and a validation set (*n* = 50) at a ratio of 7:3. Morphological features of the cerebral cortex were extracted with FreeSurfer software. Network features were extracted from gray matter with the GRETNA toolbox. The network, morphology, network-clinical, morphology-clinical, morphology-network and morphology-network-clinical models were developed by multivariate Cox proportional hazard model. The performance of each model was assessed by the concordance index (C-index).

**Results:**

In the training group, the C-indexes of the network, morphology, network-clinical, morphology-clinical, morphology-network and morphology-network-clinical models were 0.834, 0.926, 0.915, 0.949, 0.928, and 0.951, respectively. The C-indexes of those models in the validation group were 0.765, 0.784, 0.849, 0.877, 0.884, and 0.880, respectively. The morphology-network-clinical model performed the best. A multi-predictor nomogram with high accuracy for individual AD prediction (C-index = 0.951) was established.

**Conclusion:**

The early occurrence of AD could be accurately predicted using our morphology-network-clinical model and the multi-predictor nomogram. This could help doctors make early and personalized treatment decisions in clinical practice, which showed important clinical significance.

## 1 Introduction

Approximately 0.5–0.8% of the worldwide population suffers from cognitive and behavioral deficits caused by Alzheimer’s disease (AD), and the percentage is still increasing (1). The neuropathological change in AD patients is characterized by the loss of neurons and synapses in the cortex, which leads to subtle cortical morphological changes and even anatomical atrophy (1). Mild cognitive impairment (MCI) is described as a stage of transition between a normal state and the pathological state of AD, with 10 to 20% of individuals progressing to dementia within a few years (2). Many studies have shown that if AD could be predicted early and intervention was taken, the disease progression in MCI patients might be slowed or reversed (3, 4). Thus, developing an accurate method to predict the development of AD in MCI patients has important clinical implications.

In previous studies, MCI patients were usually divided into two groups based on whether they progressed to AD, and binary classification was performed (5–8). These methods did not provide specific information about the time when AD occurred. In recent years, some studies have begun to use MRI technology to predict the time of MCI conversion to AD (9–11). However, the MRI indicators used in these studies were mostly large-scale markers of brain atrophy, such as cortical volume and thickness (9–11). The characteristics of the interrelation between cortical regions were ignored. In fact, the subtle cortical changes at the early stage caused by accumulated harmful metabolites in neurons might be beyond the sensitivity of morphological indicators, which might be revealed by network indicators from gray matter covariation or similarity (12). On the other hand, the classical gray matter covariant network, which was composed of the covariance of gray matter measurements among participants, was only used for group-level brain network analysis and cannot be used for personal prediction analysis previously. The individual-level gray matter network based on brain region morphological similarities (13) has been successfully applied in the diagnosis of neurological and psychiatric disorders such as AD (14) and schizophrenia (15). Previous studies have found a close correlation between cognitive impairment in AD patients and topological randomization of the gray matter network (16). This study was the first to use this method to study the cognitive dynamics of MCI patients. We analyzed the individual cortical morphology and gray matter network characteristics of MCI patients using T1WI sequences and constructed an artificial intelligence model to achieve early prediction of AD occurrence.

## 2 Materials and methods

### 2.1 Participants

**Figure 1:**
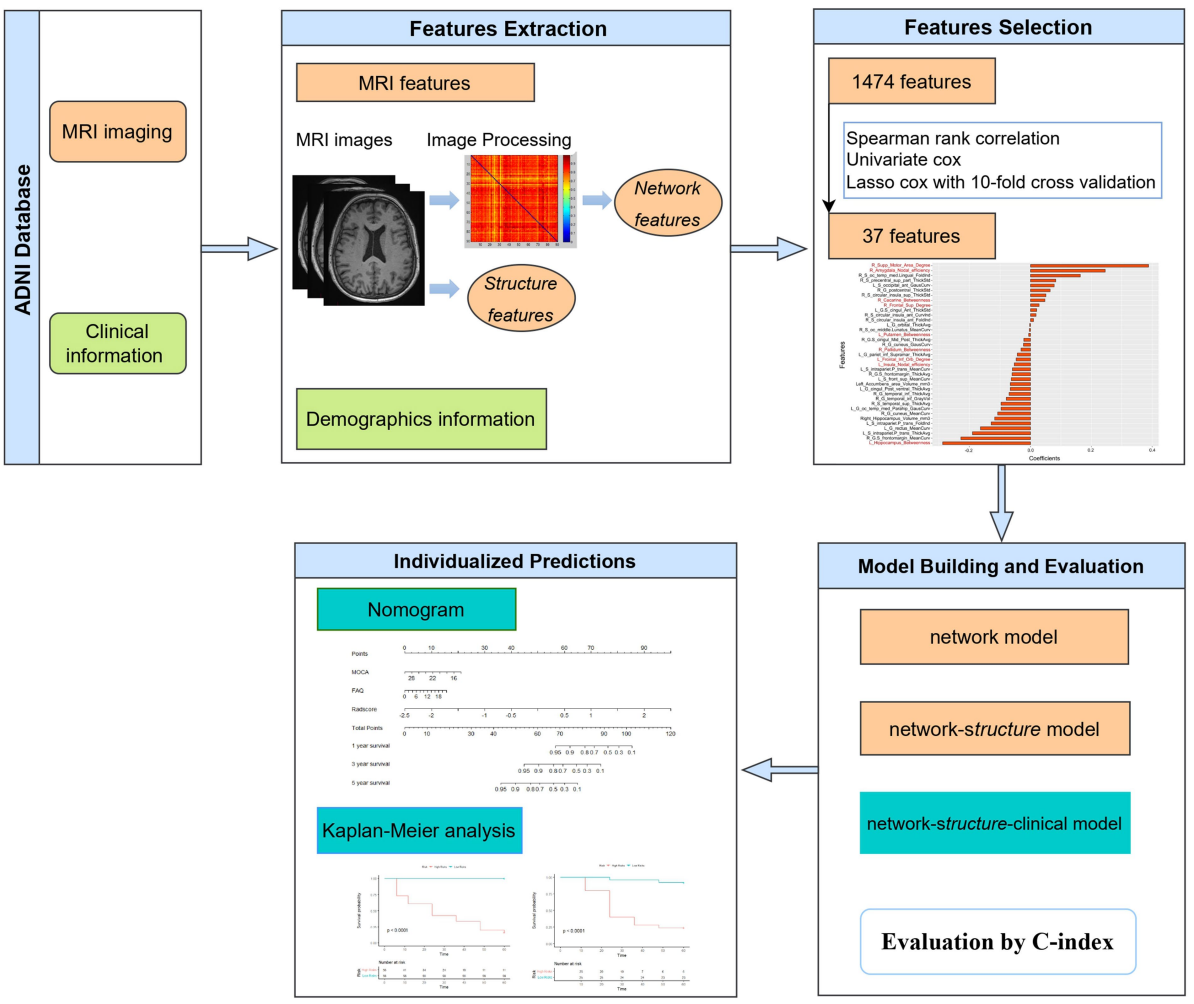
Flow chart of the study.

All participants were included from the Alzheimer’s Disease Neuroimaging Initiative (ADNI) database (adni.loni.usc.edu). The dataset included baseline T1W images and clinical data of 68 MCI patients who progressed to AD in 6–60 months and 94 other MCI patients without AD conversion in 5 years (the follow-up period was 6–12 months within 3 years and 12 months for the following 3–5 years). The diagnosis of AD followed the criteria defined by the Alzheimer’s disease and Related Disorders Association and the National Institute of Neurological and Communicative Disorders and Stroke. The MCI patients reported subjective memory concerns; however, these patients showed no significant impairment in other cognitive domains, everyday activities were substantially preserved, and there were no signs of dementia. In the follow-up period, participants with bidirectional changes (MCI to AD and back to MCI) were excluded. All patients were randomized at a ratio of 7:3 into a training group (*n* = 112) and a validation group (*n* = 50). The workflow is shown in Figure 1.

Clinical data, including age, sex, education level, body mass index (BMI), alcohol use and scores on twelve neuropsychological scales Montreal Cognitive Assessment [MOCA], 11-item and 13-item Alzheimer’s Disease Assessment Scale, Clinical Dementia Rating [CDR] Scale, Functional Activities Questionnaire [FAQ], Geriatric Depression Scale [GDS], Mini-Mental State Examination [MMSE], four subscales of the Rey Auditory-Verbal Learning Test, and Animal Fluency Test [AFT], were directly collected from the ADNI website.

### 2.2 MRI acquisition

By using a 3D-MPRAGE sequence or a comparable sequence with slightly varying resolutions, T1W images for each patient were acquired. The parameters of scanner 1 (General Electric Healthcare) were repetition time (TR) = 7.7–7.0 msec, echo time (TE) = 3.1–2.8 msec and matrix = 256 × 256 × 196. Scanner 2 was from Siemens Medical Solutions, and its parameters were TR = 2300.0 msec, TE = 3.0 msec and matrix = 240 × 256 × 176. The parameters of scanner 3 (Philips Medical Systems) were TR = 6.8 msec, TE = 3.1 msec and matrix = 256 × 256 × 170. For more information, please see http://adni.loni.usc.edu/methods/documents/.

### 2.3 Extraction of network features in gray matter

All network features were extracted from gray matter networks that were individually constructed and normalized with the methodology proposed by Tijms et al. (13) and Batalle et al. (17). The gray matter segmentation was performed with Statistical Parametric Mapping software. The connection was determined according to the statistical similarities of gray matter morphology. The similarities were determined by the maximum correlation between two cubes spanning multiple rotations based on the unified automated anatomical labeling parcellation template. These procedures yielded a similarity network for each individual, with connect strengths ranging from 0 to 1, and self-connections were removed. Details of network construction and normalization are provided in the Supplementary materials.

The GRETNA toolbox was used for the calculation of network properties (18). The global efficiency (E_g_), small-world scalar (*σ*), local efficiency (E_loc_), cluster coefficient (Cp), assortativity, characteristic path length (Lp), nodal betweenness, nodal degree and nodal efficiency were calculated (19, 20). Detailed explanations of the network properties and calculation procedures are provided in the Supplementary materials.

### 2.4 Extraction of morphological features of the cortex

Morphological features of the cerebral cortex were extracted with FreeSurfer software. First, the T1W images were normalized to the MNI space. The Brainnetome Atlas was resliced to the conventional MNI space. Then, the Destrieux atlas was used to divide the entire cortex (21, 22). Motion correction, cranial stripping, gray/white matter segmentation, and cortical surface model reconstruction were performed (23). The cortical indicators of mean thickness, surface area, standard deviation of the thickness, integrated rectified Gaussian curvature, intrinsic curvature index, integrated rectified mean curvature, folding index and gray matter volume were obtained. Subcortical brain region segmentation was carried out using SPM12 and the Brainnetome fMRI Toolkit. The gray matter volumes of the bilateral thalamus, putamen, hippocampus, caudate nucleus, globus pallidus, nucleus accumbens and amygdala were obtained.

### 2.5 Feature selection and label construction for radiomics

**Figure 2:**
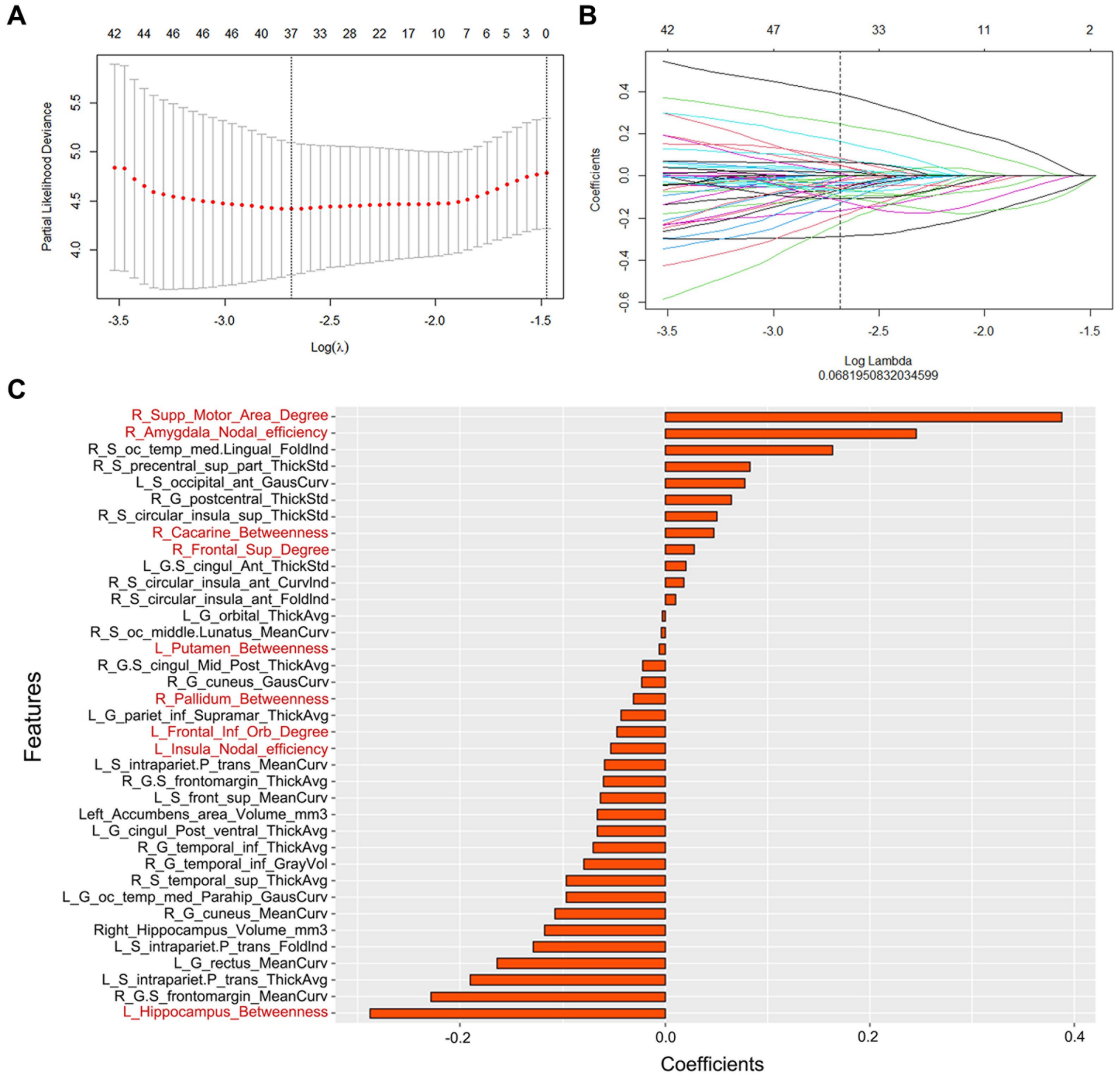
**(A)** Parameter selection in the LASSO Cox regression model. **(B)** LASSO coefficient analysis of the radiomics features. **(C)** The 37 individual radiomics features and their coefficients. Fonts in black are structural features, while fonts in red are network features.

In this study, 276 features of the gray matter network (6 global properties and 270 nodal network properties) and 1,198 features of the cortical morphology were obtained. First, redundancy was reduced by calculating the correlation coefficients between features using Spearman correlation analysis, and after removing highly correlated features, 328 features with correlation coefficients above 0.9 remained. Second, the 37 features with significant differences (*p* < 0.05) were chosen using univariate Cox analysis. Third, the most significant features in disease progression were chosen using LASSO Cox regression with 10-fold cross validation. Then, 37 radiomics features were finally determined, with 28 morphological features and 9 network features (Figure 2). The coefficients of the features were presented in Figure 2. The radiomics scores (rad-score) of each subject were calculated by combining the selected features linearly and multiplying the outputs by the corresponding coefficients. A univariate Cox proportional hazards model was utilized to identify potential predictors among the clinical parameters. The factors that were significant in the univariate analysis (*p* < 0.05) were used in a multivariate Cox proportional hazards model to establish network, morphology, network–clinical, morphology–clinical, morphology–network and morphology–network–clinical models. Finally, the consistency index (C-index) was used to assess the performance of the models in the training cohort, and the results were independently verified in the validation cohort.

### 2.6 Multi-predictor nomogram construction

In this study, multivariate Cox analysis was used to establish a multi-predictor nomogram including radiomic and clinical metrics. Calibration curves were used to analyze the correspondence between the real outcomes and the predicted results.

### 2.7 Statistical analysis

The statistical analysis was performed by R software (v. 3.6.0; http://www.Rproject.org). The Shapiro–Wilk test and Bartlett test were used for clinical, network, and morphological data analysis. The between-group differences were examined by Student’s *t* test, the Mann–Whitney U test or the chi-squared test. The C-index with a 95% confidence interval (CI) was used to assess the model performance. The Akaike information criterion (AIC) was used to estimate the potential risk of overfitting. The intraclass correlation coefficient (ICC) was adopted to evaluate interobserver reproducibility. Two-tailed *p* < 0.05 was considered statistically significant.

## 3 Results

### 3.1 Demographics

A total of 112 patients (71 males and 41 females) were included in the training group, and 50 patients (25 males and 25 females) were included in the validation group. In the training group, the average age was 72.00 ± 6.58 years, the average BMI was 27.98 ± 5.21, and the average years of education was 16.29 ± 2.63 years. In the validation group, the average age was 71.47 ± 7.82 years, with an average BMI of 27.48 ± 4.79 and an average of 15.86 ± 2.63 years of education. There was no significant difference in demographic data between the two groups (*p* > 0.05, Table 1).

**Table 1:** Baseline characteristics of all the MCI patients.

Variable	Training set (*n* = 112)	Test set (*n* = 50)	Statistics	*p* value
Sex				
1	71 (63.39%)	25 (50.00%)	2.568	0.109
2	41 (36.61%)	25 (50.00%)		
Drink				
0	107 (95.54%)	50 (100.00%)	1.053	0.305
1	5 (4.46%)	0 (0.00%)		
Age	72.00 ± 6.58	71.47 ± 7.82	−0.413	0.681
Education	16.29 ± 2.63	15.86 ± 2.63	−0.971	0.333
BMI	27.98 ± 5.21	27.48 ± 4.79	−0.583	0.561
MOCA	23.00 (21.00, 25.00)	23.00 (20.00, 25.00)	−0.477	0.634
ADAS_11	9.00 (6.45, 12.00)	9.00 (6.00, 13.05)	0.196	0.845
ADAS_13	15.00 (11.00, 20.00)	16.00 (10.95, 21.05)	0.734	0.463
CDR	0.50 (0.50, 0.50)	0.50 (0.50, 0.50)	−0.203	0.839
FAQ	2.00 (0.00, 5.00)	2.00 (0.00, 8.00)	0.502	0.616
GDSCALE	2.00 (1.00, 2.00)	1.00 (1.00, 3.00)	0.524	0.6
MMSE	28.00 (27.00, 29.00)	28.00 (26.95, 30.00)	0.276	0.783
RAVLT_immediate_bl	34.00 (28.00, 41.00)	33.50 (26.00, 41.05)	−0.259	0.795
RAVLT_learning_bl	4.00 (2.00, 6.00)	4.00 (3.00, 6.00)	0.78	0.436
RAVLT_forgetting_bl	5.00 (3.00, 6.55)	5.00 (4.00, 7.05)	0.906	0.365
RAVLT_perc_forgetting_bl	65.16 (38.00, 90.15)	75.00 (36.36, 100.00)	1.012	0.312
Category_Fluency_Animals.	17.40 ± 4.78	16.96 ± 5.47	−0.519	0.604
WM volume	4.88 (2.02, 13.28)	4.47 (1.61, 11.39)	−0.558	0.577
Fazekas	−0.27 (−0.27, −0.27)	−0.27 (−0.27, −0.27)	−0.678	0.498
Radscore	−0.13 (−0.84, 0.89)	−0.09 (−0.88, 0.82)	−0.007	0.994

MCI, mild cognitive impairment; BMI, body mass index; ADAS, Alzheimer’s Disease Assessment Scale; CDR, Clinical Dementia Rating; FAQ, Functional Assessment Questionnaire; GDSCALE, Geriatric Depression Scale; MMSE, Mini-Mental State Examination; RAVLT, Rey Auditory-Verbal Learning Test; WM, white matter.

### 3.2 Model establishment and verification

**Figure 3:**
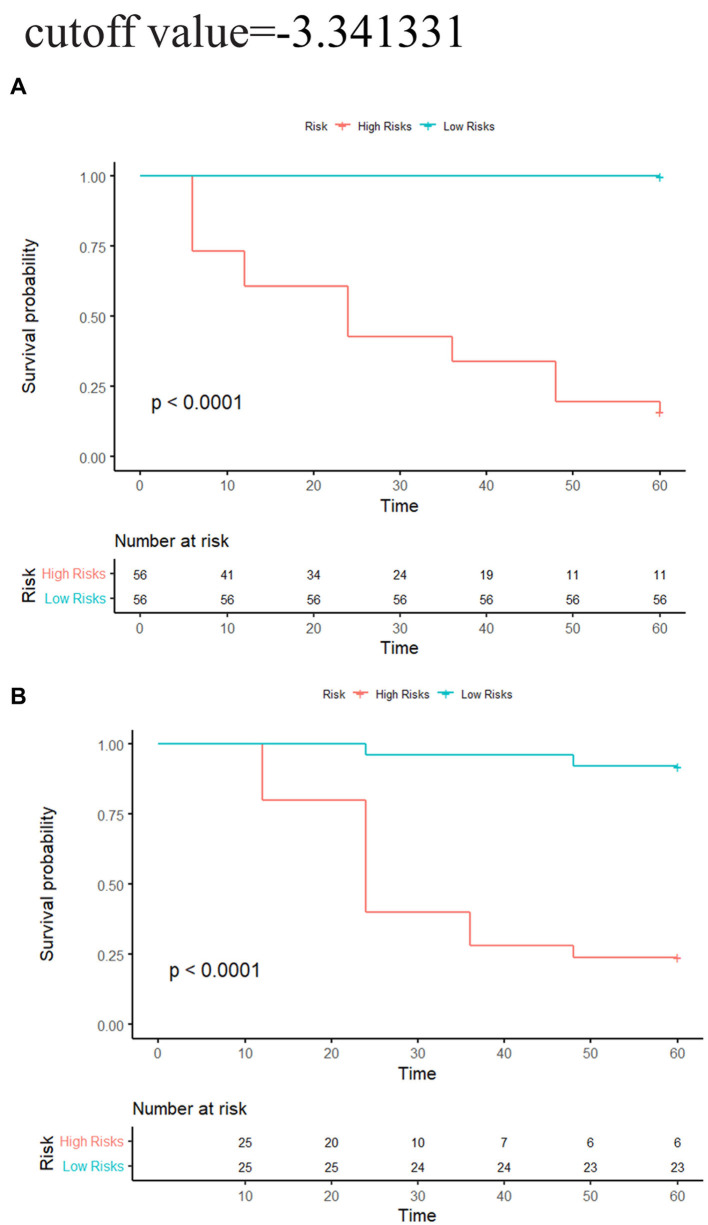
Network-morphology-clinical model-based KM analysis of the prediction with median cutoff values. **(A)** Training cohort; **(B)** validation cohort. Significant variations were observed between the low-risk and high-risk groups (log-rank test, *p* < 0.05).

In the training group, the C-indexes of the network, morphology, network-clinical, morphology-clinical, morphology-network and morphology-network-clinical models were 0.834, 0.926, 0.915, 0.949, 0.928, and 0.951, respectively. The C-indexes of those models in the validation group were 0.765, 0.784, 0.849, 0.877, 0.884 and 0.880, respectively. The morphology-network-clinical model performed the best (Table 2). The morphology-network-clinical model-based Kaplan–Meier (KM) survival curve is presented in Figure 3. The median value used for stratification of risks was 3.341331, which was the cutoff value of the morphology-network-clinical model. A substantial difference in time to progression (TTP) was observed between the low-risk and high-risk groups.

**Table 2:** Performance of the different models.

	Training set			Test set		
Models	C-index	Lower	Upper	C-index	Lower	Upper
Network	0.834	0.776	0.892	0.765	0.672	0.857
Morphology	0.926	0.896	0.957	0.784	0.672	0.896
Morphology-Clinical	0.949	0.921	0.978	0.877	0.808	0.945
Network-Clinical	0.915	0.888	0.943	0.849	0.783	0.915
Network-Morphology	0.928	0.898	0.958	0.844	0.756	0.932
Network-Morphology-Clinical	0.951	0.928	0.974	0.880	0.817	0.943

### 3.3 Multi-predictor nomogram construction and validation

**Figure 4:**
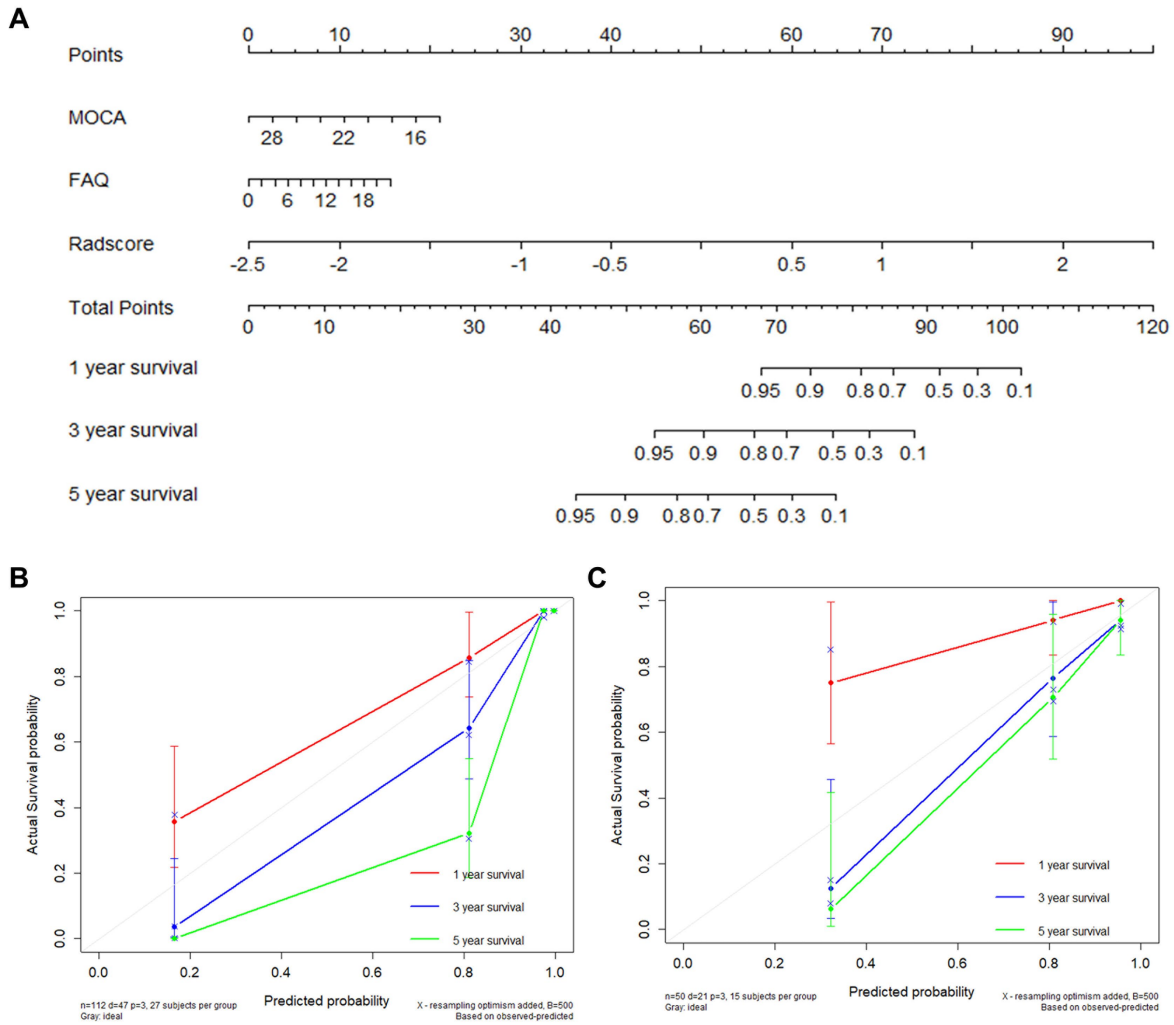
AD occurrence was estimated using the nomogram, and the model calibration was evaluated **(A)**. Calibration plot for the nomogram using the training and validation sets **(B,C)**.

A multi-predictor nomogram based on a morphology-network-clinical model was created for the purpose of individual AD prediction. This nomogram included 3 predictors: the Montreal Cognitive Assessment (MoCA) score, Functional Assessment Questionnaire (FAQ) score, and rad-score (Figure 4A). The calibration curves showed that the 1-, 3-, and 5-year predictions made by the multi-predictor nomogram and the real outcomes were in good agreement (Figures 4B,C).

## 4 Discussion

In this study, by combining cortical morphology and network features, we obtained six models for AD prediction, and all demonstrated good prognostic capability. The combined morphology-network-clinical model presented the best performance, with a C-index of 0.951 in the training group and 0.880 in the validation group, which could be regarded as an accurate predictive method to estimate the likelihood of progression in patients with MCI within a specific time frame. This method was simple and noninvasive; moreover, it did not require the use of radiation, which is of great significance in clinical practice, as radiation presents risks. Most previous studies on MCI to AD conversion only focused on the large-scale indicators of the hippocampus and temporal cortex (9–11, 24). For example, a deep learning algorithm with a C-index of 0.864 has been created to predict the early occurrence of AD based on hippocampal MRI (7). According to the thickness of the middle temporal cortex and the volume of the hippocampus, a model has been established to predict the 3-year conversion rate with a C-index of 0.78 (9). Compared with these studies, our model comprehensively analyzed the microscopic changes in the entire cerebral cortex and network for the first time and obtained higher accuracy and objectivity. On the other hand, our study only used T1WI sequences, which greatly simplified the prediction parameters and greatly improved the clinical simplicity.

In the model with the best performance, 37 features of the morphology and network (9 from the network and 28 from the morphology) were retained for AD prediction. Among the 9 features of the network, 4 were betweenness, 3 were degree, and 2 were node efficiency. Betweenness can characterize the effect of nodes on information flow. Degree reflected the ability of node information communication, whereas node efficiency measured the ability to disseminate information between two nodes. Overall, these properties could reflect the ability of the brain to transmit and integrate information that was closely related to the composition of cognitive processes (25). Previous studies have found that these parameters were damaged in AD patients, which could reflect the neuropathological state (26). Most of these features were located in the limbic areas (the left insula, left putamen, right amygdala, and left hippocampus), right supplementary motor area, right pallidum, right calcarine, left pars orbitalis, and right dorsolateral superior frontal gyrus. These regions were involved in the default-mode network, the executive control network and the salience network. All these areas were considered strongly linked with human situational memory, self-projection, and cognitive management (27–30). The DMN was the first to be identified to be affected in AD (27). Connectivity disruption in the DMN at the early stages of illness was thought to be associated with early molecular pathology in AD, which evolved before the clinical onset of dementia (31). Similarly, network alterations of the SN and ECN in AD patients were also reported, which could be a sensitive neuroimaging biomarker for AD (30, 32).

In addition to network features, 28 morphological features were also retained. Among them, 12 features belonged to cortical thickness, 13 features belonged to cortical curvature, and 3 features belonged to subcortical volume. These cortical features were of great significance in characterizing cortical atrophy and brain morphology changes. In previous studies, the decrease in gray matter volume, mean curvature and cortex thickness due to the loss of neurons and synapses in the cortex and specific subcortical areas has been proven to be closely related to the conversion of MCI to AD (33–35). Moreover, we observed 28 features that were mostly located in the insular, frontal and temporal cortex, regions that play crucial roles in memory and cognition (36–38). Atrophy of these neurons was linked to the emergence of neuropsychiatric impairments in patients suffering from AD (38–41). In our previous studies (42), we reported that the characteristics of these brain regions are helpful in reflecting disease progress.

In this study, we established a nomogram for early AD prediction, which could help doctors make personalized treatment decisions in clinical practice. Compared with previous research (42), our nomogram had higher accuracy and simpler features. In the calibration curves, the estimates showed good coherence with clinical outcomes that actually occurred. Most previous studies have tracked MCI patients for 1 to 3 years (9–11, 34, 43). All individuals in our study were followed up for 5 years, which brought our model closer to the real situation. In this study, the median of the model could correctly classify individual patients into high-risk and low-risk categories. In the high-risk group, over 75% of the MCI population developed AD within 5 years. This risk stratification has considerable value for the early clinical detection and timely treatment of high-risk MCI patients.

In this study, we utilized artificial intelligence and cortical features on T1WI sequences to establish a radiomics model and multi-predictor nomogram, which could accurately predict the early occurrence of Alzheimer’s disease. According to the median output of the joint model, MCI patients could be divided into stable and progressive subgroups, which would help identify high-risk individuals and enable them to receive timely treatment. There were several limitations in our study. First, being derived from a multicenter cohort, the ADNI database contains data from many hospitals in the United States and Canada, and heterogeneity between scanners was inevitable. Second, our research lacked independent external validation. Third, this study did not analyze dynamic follow-up data. Finally, we used a normalization method for anatomical templates, as in other experiments, to ensure that the network size was consistent across different populations. This process might have template-dependent effects. Further research should overcome these limitations and use larger samples to examine the relationship between the dynamic changes and the occurrence of AD.

## Data Availability

Publicly available datasets were analyzed in this study. This data can be found here: adni.loni.usc.edu.
