# Supplementary material for: Early prediction of Alzheimer’s disease using artificial intelligence and cortical features on T1WI sequences
Source: Front Neurol. 2025 Mar 12;16:1552940. doi: 10.3389/fneur.2025.1552940 (PMC11938367; doi:10.3389/fneur.2025.1552940)
Supplement: Supplementary file 1 [file Supplementary_file_1.docx]

Supplementary Material

**Supplementary methods**

The E_g_ reflects network integration and mathematically equals the average inverse of Lp, while E_loc_ measures information transfer capacity of a node and its neighbors. Assortativity quantifies the extent that nodes with similar degree are connected to each other (1). The σ is a metric that reflects the relation of information transfer to network density. The Cp is a metric of network segregation, measuring the local interconnectivity of the network, while the Lp is a metric of network integration, reflecting the average of the shortest path length. Betweenness, defined as the fraction of all shortest paths in the network that passthrough a given node, characterizes the node’s effect on information flow. Degree is the number of links connected to a node, which reflects its information communication ability, whilst Ne measures the ability of one node to propagate information with its connected nodes (2).

Network properties were calculated at the range of sparsity (S) thresholds of 0.05-0.40 with an interval of 0.01, and the area under the curve (AUC) was calculated for each network metric, providing a summary metric for characterizing topological properties of brain networks independent of any single threshold selection (3).

1. Newman ME. Assortative mixing in networks. *Physical review letters*. 2002;89(20):208701. doi:10.1103/PhysRevLett.89.208701.

2. Freeman LC. A Set of Measures of Centrality Based on Betweenness. *Sociometry*. 1977;40(1):35-41. doi:10.2307/3033543.

3. Zhang J, Wang J, Wu Q, Kuang W, Huang X, He Y, et al. Disrupted brain connectivity networks in drug-naive, first-episode major depressive disorder. *Biol Psychiatry*. 2011;70(4):334-42. doi:10.1016/j.biopsych.2011.05.018.

**
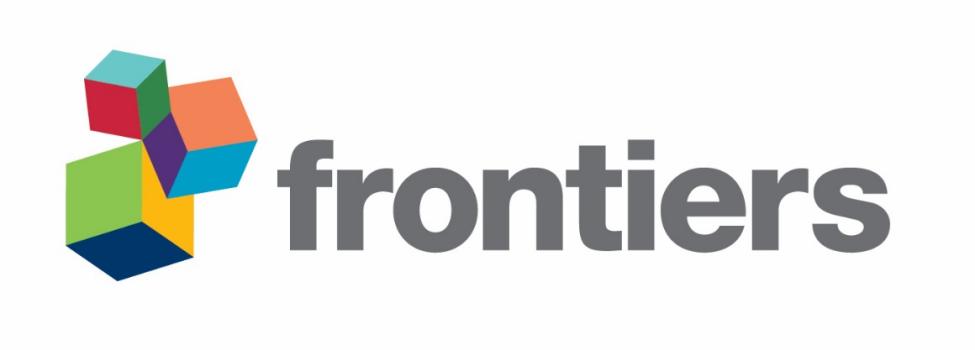
**

**Supplementary Figure 1.** The figure legends are required to have the same font as the main text, 12 point normal Times New Roman, single spaced. Please use a single paragraph for each legend and prepare the figures keeping in mind the PDF layout.
